# Microencapsulation of tris(dimethylaminomethyl)phenol using polystyrene shell for self-healing materials

**DOI:** 10.1038/s41598-020-69168-8

**Published:** 2020-07-23

**Authors:** Honglin Hu, Lu Zhang, Ying Zhang, Yunhua Yang, Ruilian Yu, Jinming Wang

**Affiliations:** 1 Science and Technology on Advanced Functional Composites Laboratory, Aerospace Research Institute of Materials & Processing Technology, Beijing, 100076 China; 2 Science and Technology on Space Physics Laboratory, Beijing, 100076 China

**Keywords:** Composites, Synthesis and processing

## Abstract

The self-healing function of the polymer material has been realized by the microencapsulation technology of the healing agent. A novel microcapsule contained tris(dimethylaminomethyl)phenol (DMP-30) with polystyrene as shell material was prepared via solvent evaporation technique in a W/O/W emulsion. Two key strategies were implemented to prepare the microcapsules successfully. First, a small amount of deionized water was added into DMP-30 to form a complex, and a stable W/O emulsion was successfully prepared. The second one is to form a stable W/O/W emulsion system with the high viscosity aqueous solution added with Arabia gum and surfactants as the third phase. In addition, the influencing factors of microcapsules preparation were investigated systematically. The chemical structure of DMP-30 microcapsule was investigated by Fourier transform infrared. The morphology and shell thickness of the microcapsules were observed by optical microscope and scanning electron microscope. The reactivity of the core material was studied by differential scanning calorimetry. The thermal properties of microcapsules were studied by thermogravimetric analysis. The environmental resistance of microcapsules was verified by the isothermal aging test. Results showed that DMP-30 was successfully coated by polystyrene and the microcapsule size was in the range of 2–40 μm. The synthesized microcapsules were thermally stable below 50 °C.

## Introduction

Thermosetting polymeric structural composites are increasingly applied in the field of aeronautics and astronautics, automobile industry, machinery industry, sports equipment, etc., which is owing to their excellent performance such as high strength and stiffness, low weight, and environmental stability^1^. Inherent brittleness and faultiness of polymeric composites make them apt to form microcracks, propagating to failure^2^. Recently, smart materials^3–5^ inspired from the biological systems were developed to repair inside damage whenever and wherever it occurs during the lifetime of polymeric composites, which would provide a method to significantly extend the service life and reliability of polymeric structural composites^6–11^. To realize this purpose, a series of strategies have been exploited such as embedded healing microcapsule and its curing agent^12,13^, embedded dual-microcapsule including healing agent and hardener^14–16^, embedded vascular network containing healing agent^17–21^, and so on^22–24^. All of these methods aim to provide a carrier for the healing agent so as to maintain its reactivity and release while the microcrack occurs. For the key to achieving the self-healing function, the healing agent should be protected from the external environment.

It is a general approach that healing agent is microencapsulated by the polymeric shell material via interfacial polymerization^25^, situ polymerization^26–29^, or solvent evaporation^30^ in a stable emulsion system. The endo-dicyclopentadiene microcapsule was firstly reported for self-healing material, which was embedded together with the Grubby catalyst in an epoxy matrix. Compared with the original epoxy resin, the average fracture toughness recovery is 60%^12^. Subsequently, as a reactive monomer, epoxy resin can react with various curing agents such as amines and anhydrides at different temperatures. Therefore, the binary self-healing composite with epoxy and hardeners attracts wide attention. Microencapsulation of epoxy was successfully synthesized firstly by Yuan via situ polymerization^26^. The curing agent of epoxy resin mainly includes reactive curing agent and catalytic curing agent. Microencapsulation of reactive curing agents for the epoxy self-healing system has been attempted with only modest success^31^. Several strategies for the microencapsulating reactive hardeners had been reported. Typically, hollow microcapsule with poly(urea–formaldehyde) shell was put into a vacuum tank filling with diethylenetriamine. Diethylenetriamine-containing microcapsules were obtained after the vacuum filtration process^22^. Jin et al.^14^ reported a binary self-healing material consisted of polyoxypropylenetriamine capsules and epoxy capsules, which satisfied the rigorous requirements for structural polymer composites cured at rising temperatures. The polyoxypropylenetriamine microcapsules were obtained by the method of vacuum infiltration. Hollow microcapsule with poly(urea–formaldehyde) shell was immersed into polyoxypropylenetriamine. Diuron-containing capsule with polystyrene (PS) shell were prepared by the method of solvent evaporation in an oil-in-water emulsion^30^. The cationic catalytic curing agent (C_2_H_5_)_2_O·BF_3_ microcapsules for self-healing material were synthesized via infiltrating method^32^. Microencapsulation of an anionic catalyst curing agent of epoxy had been rarely reported.

Compared with the reactive curing agent, catalytic curing agent doesn’t participate curing reaction and isn’t consumed by epoxy functional groups. It needs only a small amount to cure the epoxy resin. Catalytic curing agent includes cationic catalyst via cation initiated ring-opening polymerization and anionic catalyst via anion initiated ring-opening polymerization of epoxy. Tris(dimethylaminomethyl)phenol (DMP-30) is an anionic catalyst and it can also act as an effective promoter of the epoxy curing system. Therefore, DMP-30 is an ideal embedment hardener or promoter for binary self-healing epoxy. In practice, however, microencapsulation of DMP-30 is quite difficult due to its solubility in water and many organic solvents. To the authors’ knowledge, encapsulated anionic catalyst DMP-30 for self-healing materials has not been reported yet. In this work, two key strategies were implemented to prepare the microcapsules successfully. The first one is that a small amount of deionized water is added to DMP-30 to form complex, and stable emulsion of water-in-oil is prepared successfully. The second one is that high viscosity water solution prepared by adding Arabic gum and surfactant is used as the third phase so as to form a stable W/O/W emulsion system.

In this study, DMP-30-containing microcapsules were prepared successfully by solvent evaporation in a W/O/W emulsion. The influence of preparation condition on the properties of microcapsules was systematically investigated by orthographic factorial design of three factors and three levels focused on the results of average core content, diameter, and shell thickness. The optimum preparation parameters of microcapsules were concluded finally. The chemical structure and core reactivity of microcapsules were confirmed and further proved that core material was microencapsulated successfully by shell material. To provide the performance of microcapsules for making binary self-healing composites in the subsequent works, the properties including surface morphology, shell thickness, size distribution and average diameter, thermal stabilization, isothermal aging, and interfacial properties were investigated. The performance parameters of microcapsules for fabricating self-healing composites are concluded finally.

## Materials and methods

### Materials

DMP-30 used as core material was purchased from Jinan Changyingda Chemical Co., Ltd., China. Styrene used as shell material former was obtained from Sinopharm Chemical Reagent Co. Ltd., China. Dehydrated sorbitol monooleate polyoxyethylene ether (Tween-80), dehydrated sorbitol fatty acid ester (Span-80), sodium dodecyl sulfate (SDS) and sodium dodecyl benzene sulfonate (SDBS) used as a surfactant were purchased from Sinopharm Chemical Reagent Co. Ltd., China. Arabic gum and poly(vinyl alcohol) (PVA) used as stabilizers were purchased from Tianjin kemio Chemical Reagent Co., Ltd., China. Azodiisobutyronitrile (AIBN) was purchased from Nanjing Taimanniu Chemical Co., Ltd., China. Dichloromethane used as an oil phase was purchased from Sinopharm Chemical Reagent Co. Ltd., China. NaOH and Na_2_SO_4_ were purchased from Sinopharm Chemical Reagent Co. Ltd., China.

### Preparation of shell material polystyrene (PS)

Styrene purification: 250 ml styrene was added in a 500 ml separating funnel, rinsed with 50 ml of 5wt% NaOH aqueous solution and deionized water for several times, respectively. Then anhydrous Na_2_SO_4_ was added into styrene for drying. After that, styrene was distilled and the fraction was collected at 60 °C (5.33 KPa). Pure styrene was obtained.

36 ml pure styrene and 0.36 g ANIB were mixed in a 500 ml three-neck round-bottomed flask with mechanical stirring. 0.3wt% PVA and 200 ml deionized water were added into the mixture. The temperature of the system was raised to 90 °C and kept for 2–3 h. The system was chilled down to room temperature, and centrifugal separation for 15 min. The solid PS was obtained after rinsed with deionized water, after that, dried at 50°Cfor 2 h in an oven.

### Preparation of microcapsules

Dichloromethane and shell material PS were mixed in a 250 ml three-neck round-bottomed flask with mechanical stirring at room temperature. The weight ratio of dichloromethane to polystyrene is 1:1. 0.8wt% Span-80 and 1wt%SDBS were added into the mixture. Core material composed of DMP-30 and deionized water was added into the mixture so as to form a water-in-oil emulsion under the agitation ratio of 250–350 rpm for 15–30 min. A small amount of deionized water was added to DMP-30 to form a complex, which can promote the stabilization of W/O emulsion. The weight ratio of DMP-30 and deionized water is 10:1. Then the emulsion was dropwise added into the third phase consisted of deionized water and 4wt% stabilizer under the agitation ratio of 250–350 rpm, which forms a stable W/O/W emulsion system. The system was heated to 35 °C for 4 h. The microcapsules were filtered and air-dried for 24 h. An orthographic factorial design was designed in Table 1.

**Table 1:** Creation of three factors and three levels of orthogonal factor design for the preparation of microcapsules.

Level	Factor		
	Weight ratio of core/shell A	Agitation rate (rpm) B	Emulsifier for the third phase (weight ratio) C
1	1.4:1	250	SDS:PVA = 3:1
2	1.6:1	300	SDS:Arabic gum = 3:1
3	1.8:1	350	Tween80

### Preparation of epoxy with DMP-30-containing microcapsules

TETA and DGEBPA with the weight ratio of 13:100 were mixed in a beaker. Then DMP-30 capsules were added to the resin under stirring for 1 h. To remove the inside bubbles, the resin mixture was placed in a vacuum oven at room temperature. Subsequently, the epoxy with DMP-30-containing capsules was cured at 30°Cfor 72 h, then cured at 50 °C for 8 h. The epoxy sample with microcapsules was obtained after natural cooling.

### Characterization

The observation of shell thickness and morphology of capsules was performed by SEM (QUANTA 200 ESEM, FEI) and OM (BX51, OLYMPUS). The average diameter and size distribution of microcapsules were measured by SEM observation. At least 200 measurements were carried out. The chemical structure of microcapsule was identified by FTIR spectrometer (AVATAR 370 THERMO NICOLET). The reactivity of core material was analyzed by DSC (Setaram DSC 141). Samples were heated in N_2_ at a rate of 10 °C·min^−1^ from 30 to 250 °C. The samples were prepared by the method of potassium bromide tableting. The thermal stabilization of microcapsule was investigated by TGA (Pyris 6). The microcapsule samples were heated in N_2_ at a rate of 10 °C·min^−1^ from 30 °C to 600 °C. The isothermal aging performance of the capsule was evaluated by measuring the weight loss of capsules at 50 °C in an oven as the increase of exposure time. The core content of the capsule was measured by weighting capsules (W_0_) and the capsule shell (W_1_). The capsule shell material was prepared by grinding capsules, rinsed using deionized water, filtered, and dried at 95 ~ 105 °C for 2 h. Each sample is measured for 5 times in parallel. The content of the core material was calculated by the formula (1):

1 $$Core\;content = \frac{{W_{0} - W_{1} }}{{W_{0} }} \times 100\%$$

The structural integrality of the capsule is an important factor for fabricating composite, which is mainly decided by the shell thickness of the capsule. The two-point coordinate of wall shell was obtained by graph digitizer software from the SEM photograph. The capsule shell thickness^33^ was calculated by the two points distance formula $d = \sqrt {(x_{1} - x_{2} )^{2} + (y_{1} - y_{2} )^{2} }$. The average shell thickness of each microcapsule sample is measured on at least 5 data sets.

## Results and discussion

### Microencapsulation process

**Figure 1:**
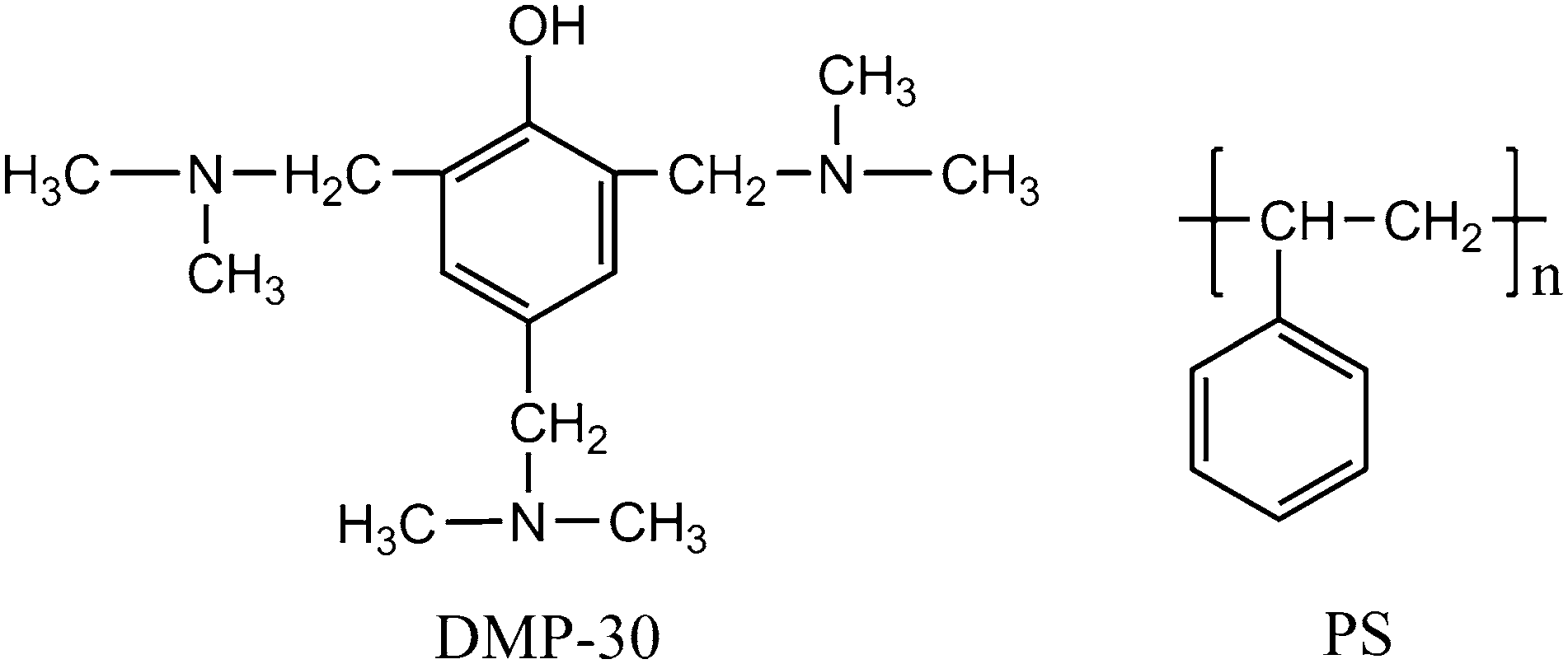
Chemical structure of core material DMP-30 and shell material PS.

**Figure 2:**
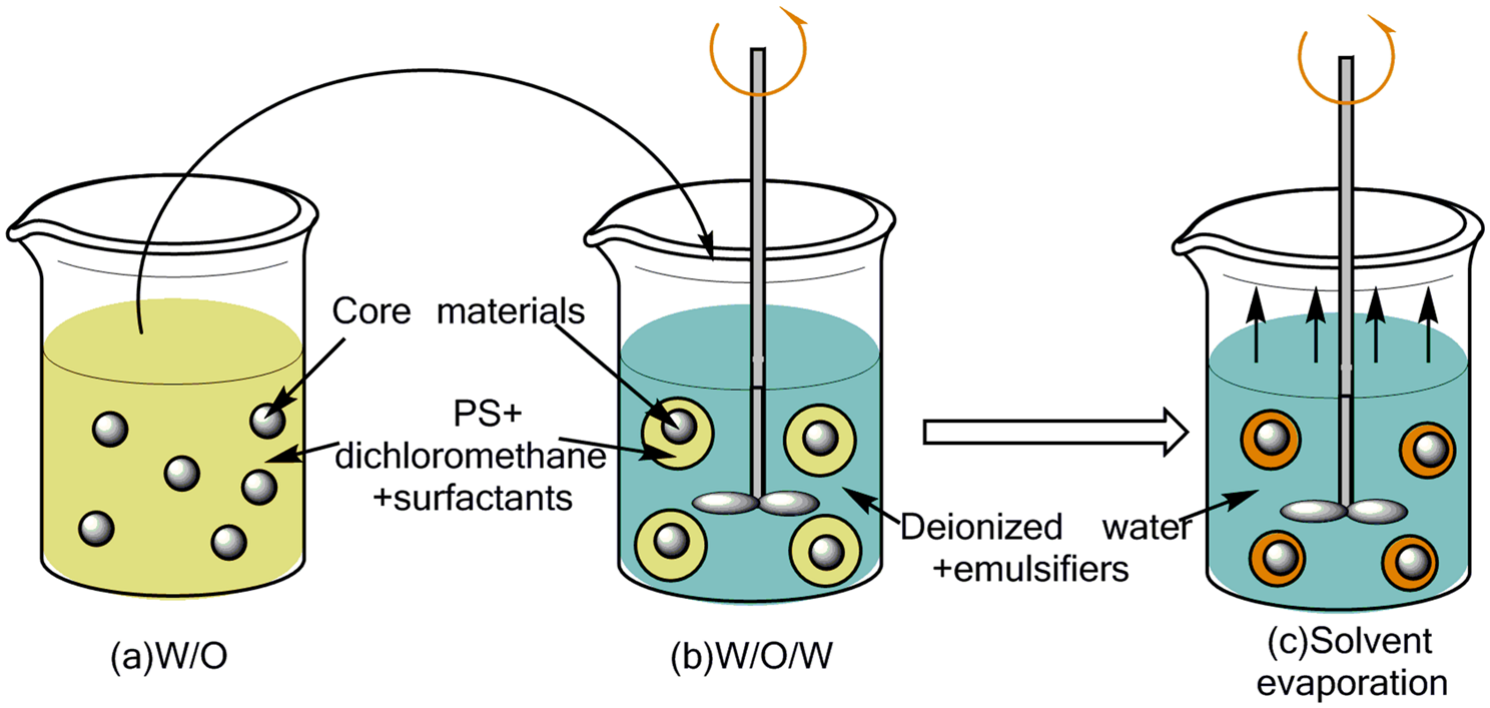
Schematic showing the preparation of DMP-30-containing microcapsules.

The chemical structures of core material DMP-30 and shell material PS are shown in Fig. 1. The preparation diagram of DMP-30 microcapsule is shown in Fig. 2. First, the PS, emulsifiers, and dichloromethane are added together to form a continuous phase. Then the core material is added to form a W/O emulsion (Fig. 2a). Second, the mixture is dropwise added to the third phase composed of deionized water and stabilizers under agitation, forming a W/O/W emulsion system (Fig. 2b). As the rise of temperature, dichloromethane is gradually evaporated, shell material depositing at the interface to form a core–shell structure (Fig. 2c).

### Influencing factors of preparation of microcapsules

PS shell microcapsules containing DMP-30 were prepared by solvent evaporation in a W/O/W emulsion. The core/shell ratio, stirring rate, and the third phase emulsifier have an influence on the properties of the microcapsules. Therefore, the influence factors of preparation of DMP-30 microcapsules were studied by designing the orthogonal factorial experiment. The core content, average diameter and average shell thickness of the microcapsules were taken as the response of the designed experiments. Table 2 shows the orthographic factorial design of the preparation parameter of microcapsule (refer to Table 1). Table 3 shows the results of the effects of the three factors at three levels on microcapsule’s performance (refer to sample No. in Table 2).

**Table 2:** Orthographic factorial design of preparation parameter of the microcapsule.

Sample no.	Factor		
	A	B	C
1	1	1	1
2	1	2	2
3	1	3	3
4	2	1	2
5	2	2	3
6	2	3	1
7	3	1	3
8	3	2	1
9	3	3	2

**Table 3:** Result of the orthographic factorial design of the microcapsule sample.

Sample no.	1	2	3	4	5	Average value	Standard deviation
**Core content (%)**							
1	14.9	14.9	15.2	15.2	15.2	15.1	0.2
2	30.3	30.1	29.7	30.3	29.9	30.1	0.3
3	3.0	3.1	3.3	3.1	3.3	3.2	0.1
4	28.9	28.6	29.0	28.9	29.2	28.9	0.2
5	3.9	3.8	3.8	3.7	3.6	3.8	0.1
6	14.3	14.5	14.6	14.5	14.4	14.5	0.1
7	4.2	4.1	4.2	4.0	4.1	4.1	0.1
8	13.5	13.4	13.6	13.5	13.7	13.5	0.1
9	29.3	28.7	28.9	29.1	29.2	29.0	0.2
**Average diameter (μm) after Gauss Fit of measured data**							
1	18	19	22	23	20	20	2
2	19	17	18	15	16	17	2
3	14	13	15	16	15	15	1
4	22	20	21	19	23	21	2
5	17	17	19	18	19	18	1
6	18	16	17	14	15	16	2
7	22	21	23	20	19	21	2
8	17	16	18	17	20	18	2
9	16	17	15	17	15	16	1
**Shell thickness (μm)**							
1	4.2	4.4	4.0	3.8	3.7	4.0	0.3
2	2.8	3.3	3.4	3.5	3.1	3.2	0.3
3	2.8	2.9	2.7	2.8	3.0	2.8	0.1
4	2.9	3.1	3.0	3.2	2.8	3.0	0.2
5	2.9	2.8	3.0	2.7	2.7	2.8	0.1
6	2.6	2.8	2.7	2.6	2.5	2.6	0.1
7	2.5	2.4	2.7	2.8	2.5	2.6	0.2
8	2.4	2.6	2.5	2.7	2.4	2.5	0.1
9	2.3	2.2	2.3	2.4	2.5	2.3	0.1

**Figure 3:**
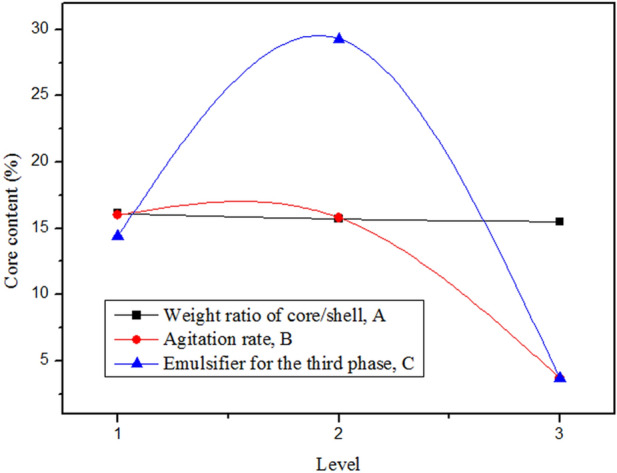
Effect of different levels of three factors (refer to Table 1) on the average core content (R_ij_ value listed in Table 3).

The content of the core material of microcapsules is a vital importance parameter for the solvent evaporation method because it can reflect the proportion of core material coated by shell material. From Table 4, according to the theory, the larger the N_i_ is, the more important the influencing factor is. Therefore, the importance of influencing factors on core content from high to low in turn is emulsifier, agitation rate and core/shell ratio. Figure 3 shows the effect of different levels of the three factors (see Table 1) on the core content (R_ij_ values listed in Table 4). The results showed that the emulsifier had a very obvious effect on the core content of the microcapsules. The emulsifier can reduce the Gibbs free energy at the interface between the oil and water phase, and keep the emulsion stability. It leads to the increase of the core content of microcapsules. As a result from the experiment based on the core content of microcapsule, the optimum preparation condition of microcapsule can be obtained from Table 3 and Fig. 3: 1.4:1 for the core/shell ratio, 250 ~ 300 rpm for the stirring rate, and emulsifier composed of SDS and Arabic gum.

**Table 4:** Analysis of the orthographic experiment of microcapsule core content (%).

No	Factor		
	A	B	C
K _i1_	48.4	48.1	43.1
K_i2_	47.2	47.4	88.0
K_i3_	46.6	11.1	11.1
R_i1_	16.1	16.0	14.4
R_i2_	15.7	15.8	29.3
R_i3_	15.5	3.7	3.7
N_i_	0.6	12.3	25.6

K_ij_ denotes the sum of microcapsule’s average core content with factor i and level j; R_ij_ denotes the average of K_ij_. R_ij_ = K_ij_/n_i_, n_i_ is the number of level j with the same factor i; N_i_ = Max(R_ij_) − Min(R_ij_), (i = A, B, C; j = 1, 2, 3)^33^.

In order to estimate the influencing factors of the average particle size of the microcapsule, the orthogonal experimental analysis of the average particle size was carried out. It can be seen from Table 5 that the importance order from high to low is stirring rate, core/shell ratio and emulsifier according to the Ni value. Similarly, it can be seen from Table 6 that the importance of factor affecting the average shell thickness is core/shell ratio, stirring rate and emulsifier in turn.

**Table 5:** Analysis of the orthographic experiment of averaged microcapsule diameter (μm).

No	Factor		
	A	B	C
K_i1_	52.0	61.0	54.0
K_i2_	55.0	53.0	54.0
K_i3_	54.0	53.0	53.0
R_i1_	17.3	20.3	18.0
R_i2_	18.3	17.7	18.0
R_i3_	18.0	17.7	17.7
N_i_	1.0	2.7	0.3

K_ij_ denotes the sum of microcapsule’s average diameter with factor i and level j; R_ij_ denotes the average of K_ij_. R_ij_ = K_ij_/n_i_, n_i_ is the number of level j with the same factor i; N_i_ = Max(R_ij_) − Min(R_ij_), (i = A, B, C; j = 1, 2, 3)^33^.

**Table 6:** Orthographic experiment analysis of averaged microcapsule shell thickness (μm).

No	Factor		
	A	B	C
K _i1_	10.0	9.6	9.1
K_i2_	8.4	8.5	8.5
K_i3_	7.4	8.2	8.2
R_i1_	3.3	3.2	3.0
R_i2_	2.8	2.8	2.8
R_i3_	2.5	2.7	2.7
N_i_	0.9	0.5	0.3

K_ij_ denotes the sum of microcapsule’s average shell thickness with factor i and level j; R_ij_ denotes the average of K_ij_. R_ij_ = K_ij_/n_i_, n_i_ is the number of level j with the same factor i; N_i_ = Max(R_ij_)  − Min(R_ij_), (i = A, B, C; j = 1, 2, 3)^33^.

### Influence of core/shell ratio on the microcapsule preparation

**Figure 4:**
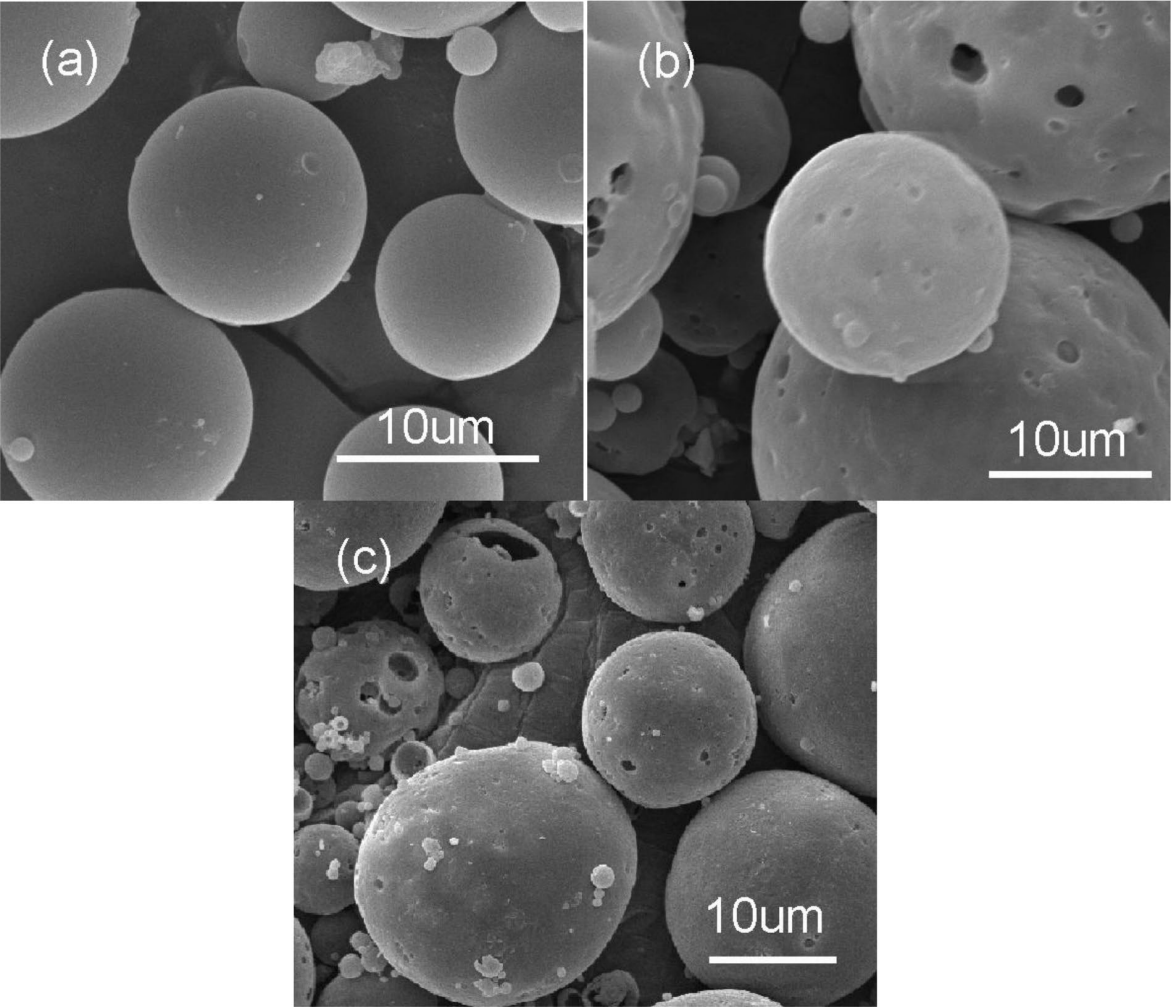
SEM photos of DMP-30-containing microcapsules at different core/shell ratios: (**a**) 1.4:1; (**b**) 1.6:1; (**c**) 1.8:1. Synthesis condition: 300 rpm for the stirring rate, and emulsifier composed of SDS and Arabic gum.

The SEM photos of DMP-30-containing capsules at varying core/shell ratio are shown in Fig. 4. As the rise of the core/shell ratio, the microcapsule surface appears holes or pores and incompletely coating by PS shell. The main reason is that when the core/shell ratio is high and other process parameters are unchanging, the shell material is not enough to encapsulate the core material, resulting in the decrease of the microcapsule core content. As a result, the optimum preparation condition can be concluded: 1.4:1 for the core/shell ratio.

### Influence of agitation rate on the microcapsule size

**Figure 5:**
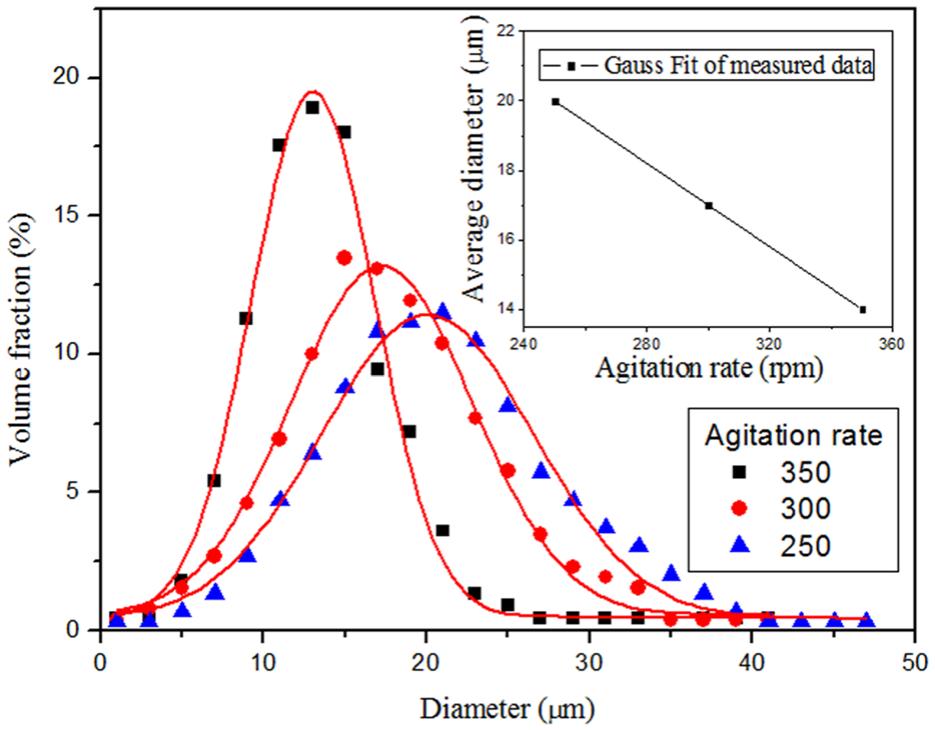
Size distribution and average diameter of DMP-30-containing microcapsules prepared at different stirring rates. Synthesis condition: 1.4:1 for the core/shell ratio, and emulsifier composed of SDS and Arabic gum.

The influence of agitation rate on average diameter and size distribution of microcapsules is shown in Fig. 5. As the decrease of stirring rate, the size distribution broadens gradually and the average diameter becomes larger. The reason is that the droplet is subjected to small shear stress at a low stirring rate, thus forming a larger microcapsule, which widens the particle size distribution. This is consistent with the results of other researchers^34,35^. These results may reflect the fact that the size of the colloid decreases with the increase of stirring speed. However, the increase of the agitation rate would destroy the colloid droplets owing to the stronger shear stress, which leads to the direct decrease of core content (Table 3: R_B1_ denotes the average core content at 250 rpm for agitation rate is 16.0%, similarly, R_B2_(300 rpm) = 15.8% and R_B3_(350 rpm) = 3.7%). Therefore, a range of adequate agitation rate can be concluded to 250–300 rpm.

### Influence of emulsifier on the microcapsule preparation

**Figure 6:**
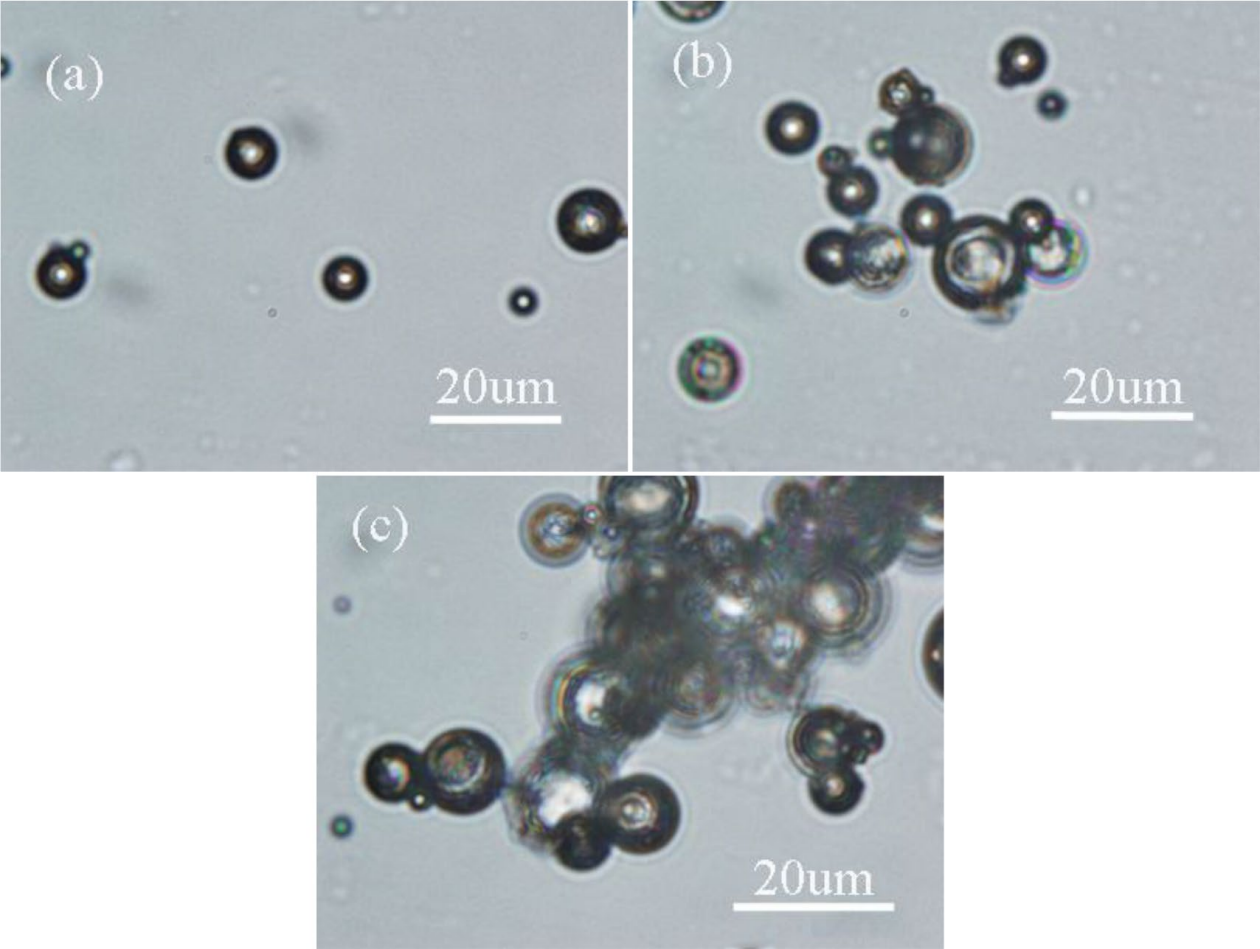
OM photos of DMP-30-containing microcapsules at different emulsifiers for the third phase (**a**) SDS and Arabic gum; (**b**) SDS and PVA; (**c**) Tween-80. Synthesis condition: 1.4:1 for the core/shell ratio, and 300 rpm for the stirring rate.

The OM photos of capsules prepared at varying emulsifiers are shown in Fig. 6. The diffraction ring can be obviously observed in all photos, which infers that the core material is successfully coated by PS shell material according to the optical theory that the diffraction ring will occur at the interface between the two different refractive indexes media^33^. However, the agglomeration among microcapsules presents differences. Agglomeration is an important property of capsules, which has an effect on the dispersion of microcapsules in the resin matrix. Compared with emulsifier composed of SDS and PVA (Fig. 6b) and single emulsifier Tween-80 (Fig. 6c), the less agglomeration among microcapsules prepared by emulsifier composed of SDS and Arabic gum can be observed from Fig. 6a.

**Figure 7:**
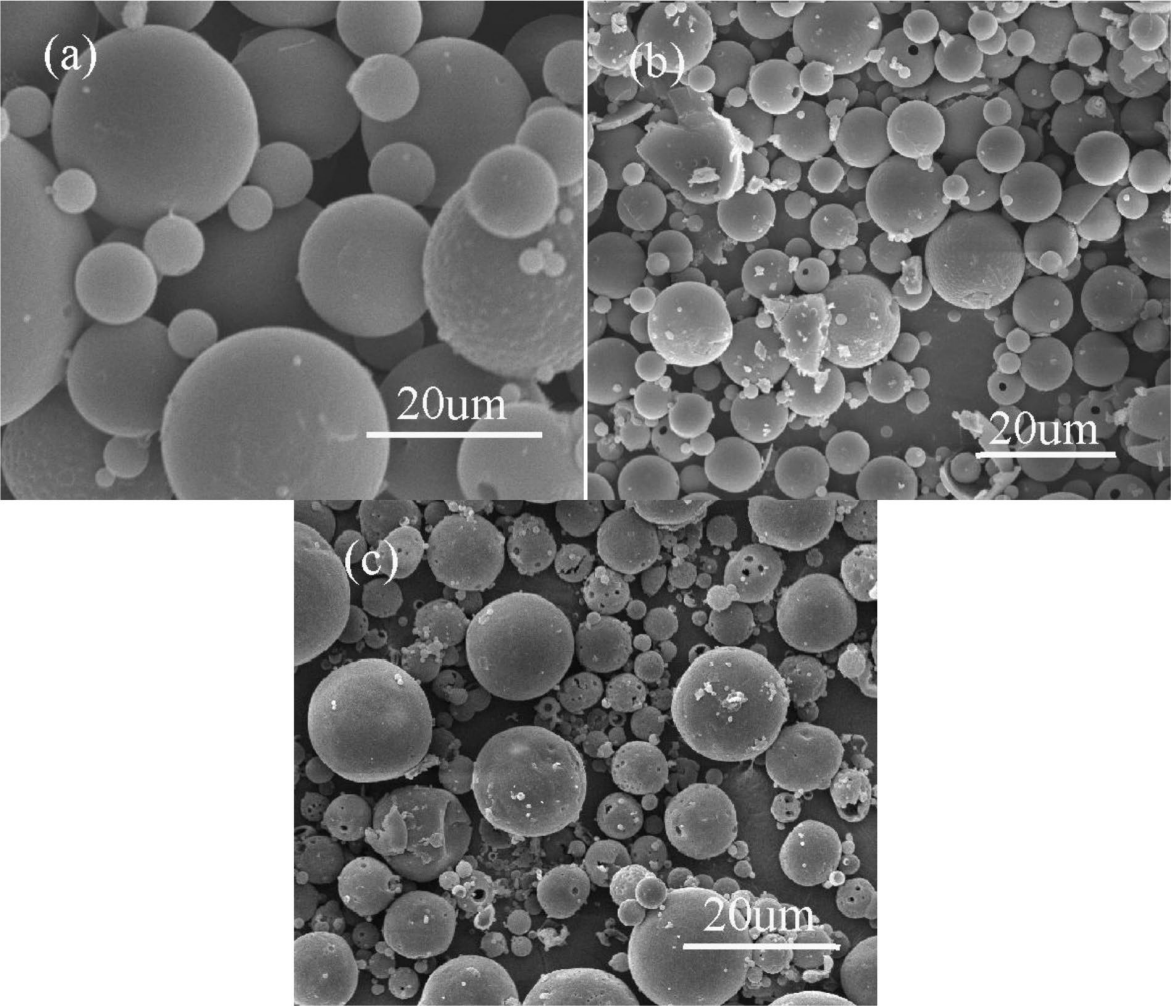
SEM photos of DMP-30-containing microcapsules at different emulsifiers for the third phase (**a**) SDS and Arabic gum; (**b**) SDS and PVA; (**c**) Tween-80. Synthesis condition: 1.4:1 for the core/shell ratio, and 300 rpm for the stirring speed.

The function of the emulsifier for the third phase is not only dispersing colloid droplets, but also stabilizing the W/O/W emulsion system. Therefore, the emulsifier for the third phase is a crucial factor for preparing microcapsules via solvent evaporation in a W/O/W system. Figure 7 shows the surface morphology of the resultant microcapsules. Although the microcapsules can be prepared at different emulsifiers, the difference in surface morphology can be obviously observed. The microcapsules prepared by emulsifier composed of SDS and Arabic gum possess smooth surface and fewer pores (Fig. 7a). Instead of emulsifier composed of SDS and PVA, the number of microcapsules with pores on the surface increases (Fig. 7b), which would result in the core material losing protection from the shell. Similarly, the microcapsules prepared by Tween-80 possess the most pores on the surface (Fig. 7c). For the reason, the emulsion system of SDS and Arabic gum is more stable than the others. Considering the agglomeration and surface morphology of microcapsules, the optimum emulsifier for the third phase can be concluded: emulsifier composed of SDS and Arabic gum.

### Microcapsule chemical structure

**Figure 8:**
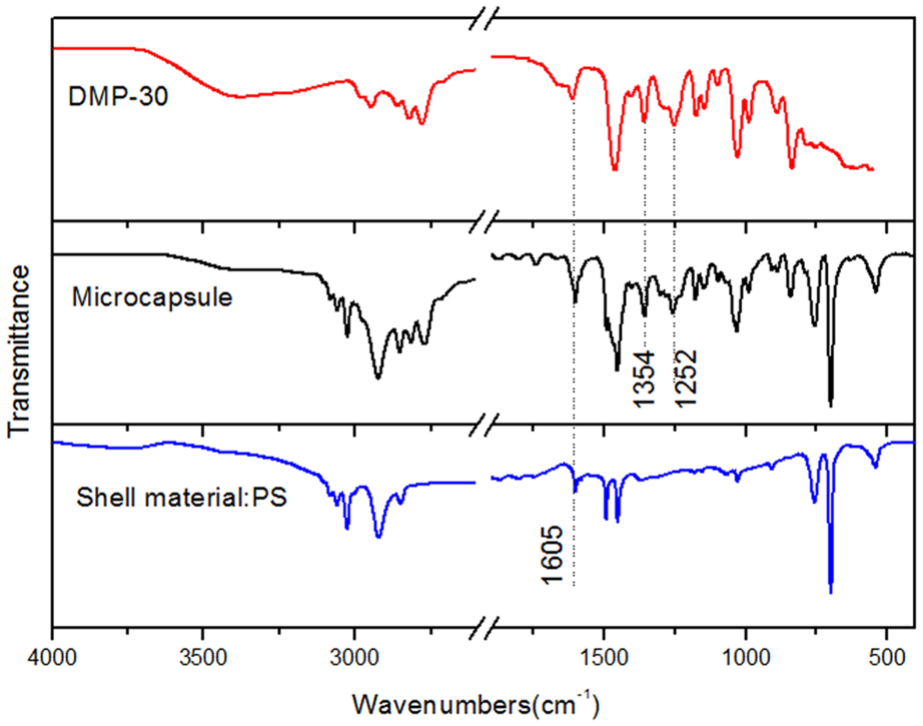
FTIR of shell material PS, DMP-30-containing microcapsules and core material DMP-30.

The FTIR spectra of DMP-30, microcapsules, PS shell material is shown in Fig. 8. The peak assignments of DMP-30 and PS are listed in Table 7. The FTIR spectrum of microcapsules containing the DMP-30 presents absorption peaks of the phenolic hydroxyl group at 1354 cm^−1^ and absorption peaks of C–OH at 1,252 cm^−1^, which indicates that the DMP-30 is successfully encapsulated by PS.

**Table 7:** Analysis of the FTIR spectra of PS and DMP-30.

PS	Approximate assignment	DMP-30	Approximate assignment
3000–3070	*ν*C–H (phenyl ring)	3400	*ν*O–H
2848–2930	*ν*C–H	2770–2970	*ν*C–H
1605	*ν*C=C (phenyl ring)	1613	*ν*C=C (phenyl ring)
		1354	*δ*O–H
		1252	*ν*C–OH

### Reactivity of DMP-30-containing microcapsule

**Figure 9:**
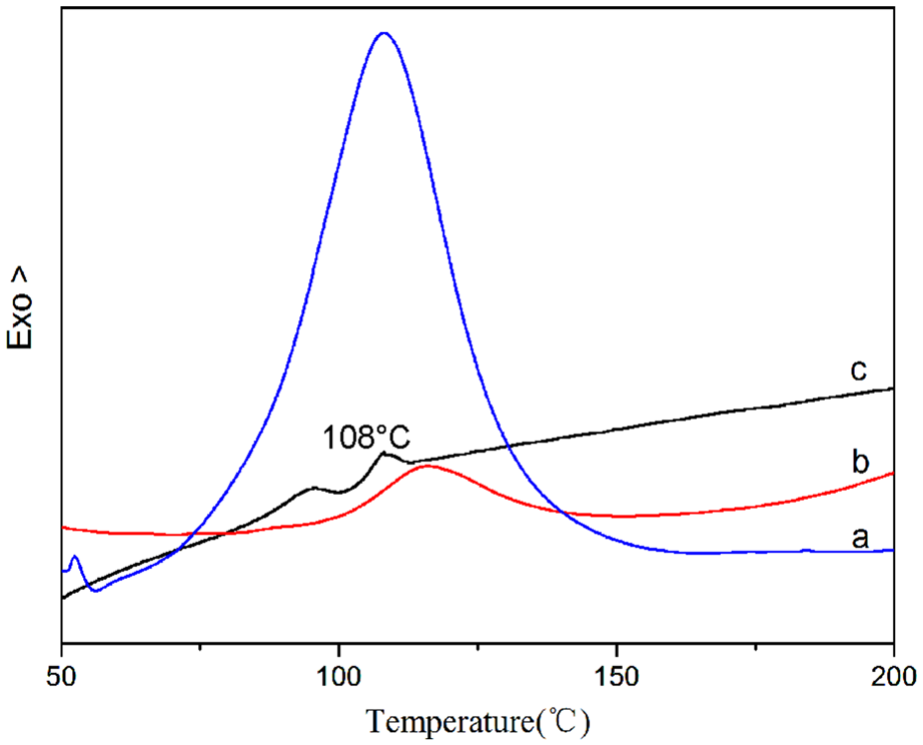
DSC curves of (**a**) DMP-30/DGEBPA = 1:10, (**b**) ground microcapsules/DGEBPA = 3.3:10, (c**)** shell material PS. The compositions are expressed in terms of weight ratios.

To evaluate the reaction activity of DMP-30 in microcapsules, DSC tests were carried out. The core content of microcapsules used in DSC tests is 30.1%. From the DSC curve of the mixture of DMP-30/DGEBPA in Fig. 9, an obvious exothermic reaction peak is detected at temperature 108.06 °C with a reaction heat of 211.70 J/g. When DMP-30 was replaced by the DMP-30-containing microcapsules, a similar exothermic peak with a reaction heat of 27.45 J/g appears at 115.83 °C. It infers that the DMP-30 in microcapsules presents reaction activity. Therefore, DMP-30-containing microcapsules possess reactivity. It indicates that DMP-30 was coated by PS via the solvent evaporation technique in a W/O/W emulsion. In addition, the glass transition temperature T_g_ of shell material PS is 108 °C from the DSC curve.

### Physical properties of microcapsules

The DMP-30-containing microcapsules were prepared under the condition of 1.4:1 for core/shell ratio, 300 rpm for the stirring speed, and 4wt% emulsifier composed of SDS and Arabic gum for the third phase. The microcapsule physical properties including shell thickness, surface morphology, average diameter, and size distribution were analyzed.

**Figure 10:**
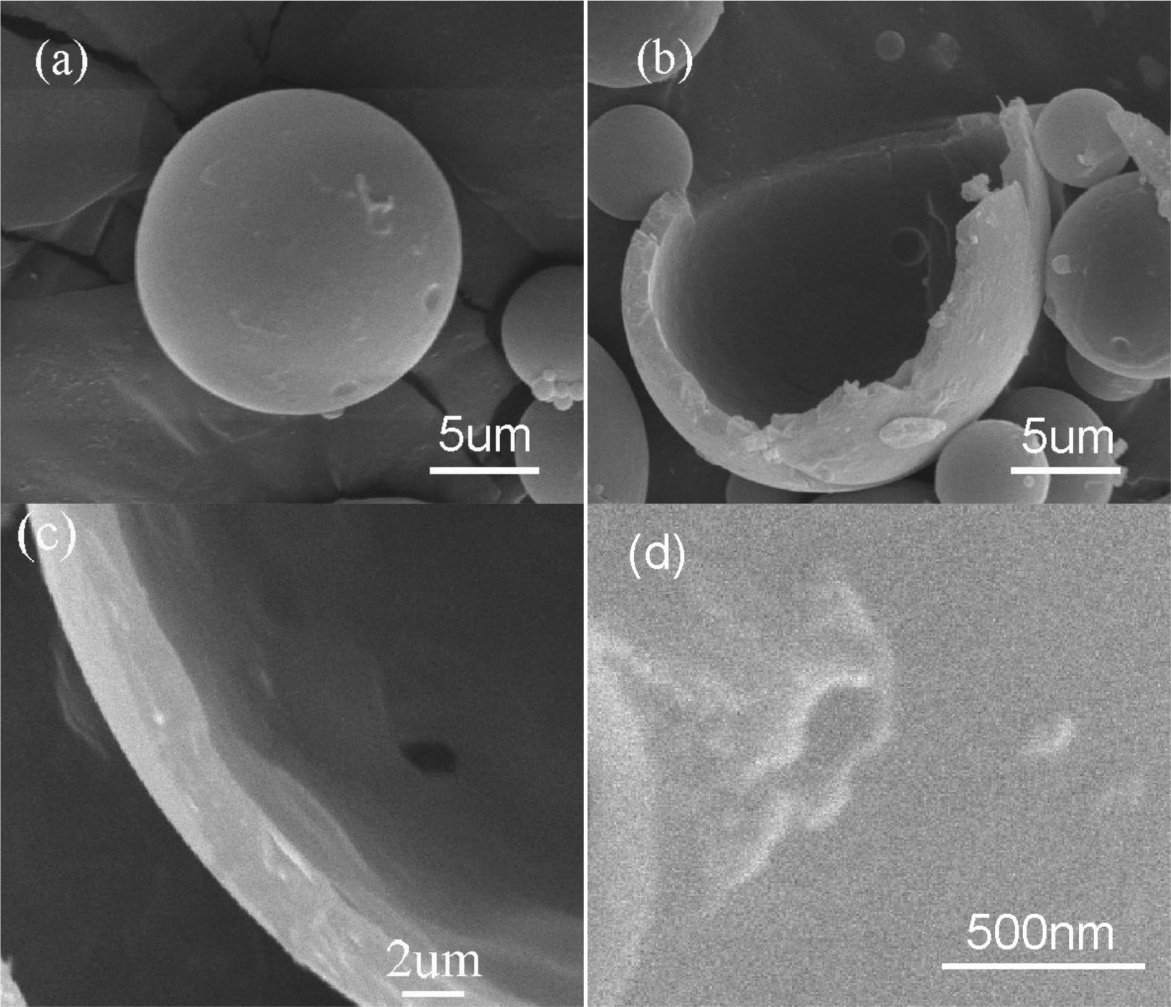
SEM photos of DMP-30-containing microcapsules, (**a**) overview; (**b**) fracture microcapsule; (**c**) shell thickness; (**d**) surface.

Figure 10 shows the SEM micrograph of the microcapsule sample. The microcapsules are spherical (Fig. 10a). The outer surface of the microcapsule is intact. There is strong evidence that the microcapsule containing DMP-30 was successfully prepared according to Fig. 10b, which shows the crack microcapsule. In Fig. 10c, the microcapsule shell thickness is 3.2 μm. It can be observed from Fig. 10d that the microcapsule surface has irregular bulges, which may be conducive to the physical connection between microcapsule and resin matrix.

**Figure 11:**
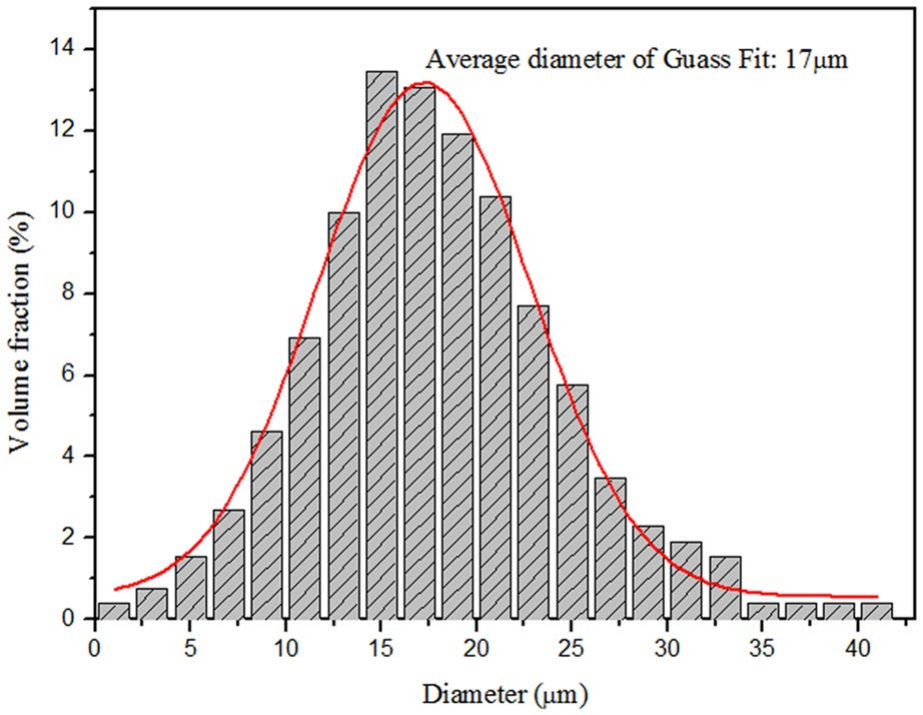
Size distribution and average diameter of microcapsules.

Figure 11 shows the average particle size and size distribution of the microcapsules. The size distribution of microcapsules is in the range of 2–40 µm and the average diameter is 17 µm. The average diameter of microcapsules prepared by solvent evaporation in a W/O/W emulsion is mainly influenced by the emulsifier and agitation rate of the third phase. The emulsifier determines the stabilization of the emulsion system. The agitation rate determines the size of the colloid droplets. The fluid flow around the impeller is turbulent. There are larger microcapsules in the area far away from the impeller, and many smaller microcapsules exist near the impeller blades. Therefore, the microcapsule diameter can be controlled by adjusting the stirring speed.

### Thermal stabilization of microcapsules

The structure integrality of microcapsules during the fabrication of self-healing materials is significant, which needs the microcapsules to possess appropriate thermal stabilization and thermal tolerance. Therefore, TGA and the isothermal aging experiment were carried out.

**Figure 12:**
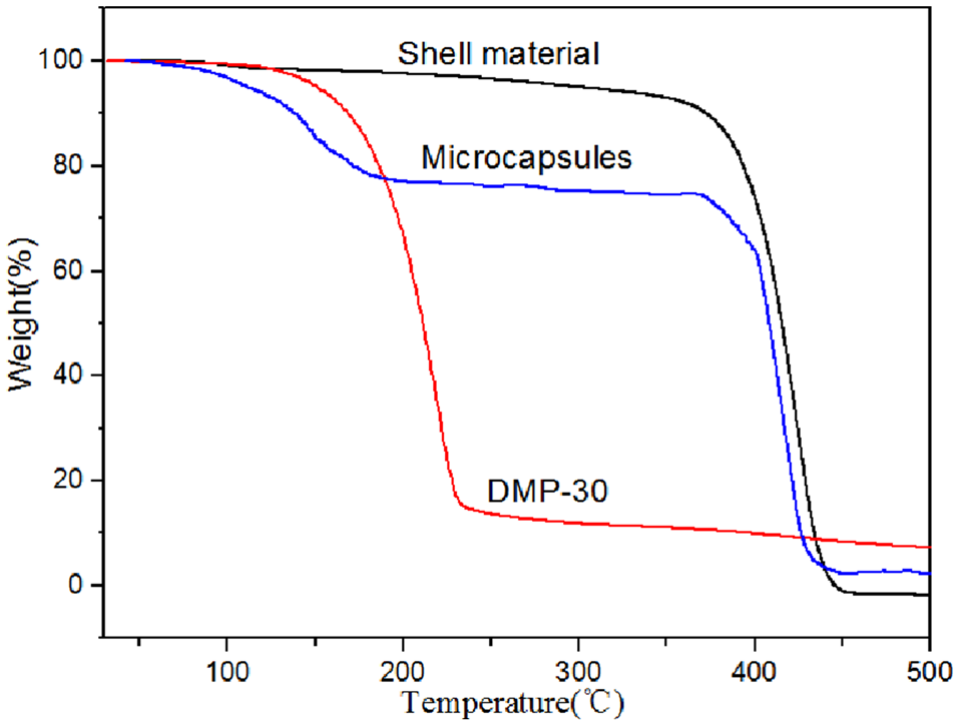
Thermal stabilization of DMP-30-containing microcapsules.

Figure 12 shows TGA curves of microcapsules, shell material PS, and DMP-30. The curve of shell material indicates that the slight weight loss before 370 °C is mainly due to the elimination of the solvent. The weight loss at temperature 370–450 °C is mainly due to the decomposition of PS. From the TG curve of DMP-30, the weight loss of DMP-30 begins at 150 °C. The curve of microcapsules includes three stages of weight loss. In the first stage from 50 to 90 °C, the weight loss is due to the evaporation of the residual small molecule. In the second stage from 90 to 180 °C, the weight loss falls faster than the first stage. The reason is that the mechanical performance of shell material decreases when the temperature reaches the glass transition temperature (T_g_ = 108 °C from curve c in Fig. 9) of PS. The shell material loses the protection function, leading to the evaporation of core material DMP-30 and water, which results in the weight loss of microcapsules. In the range of 180–370 °C, the core material of microcapsules has been completely evaporated. In the third stage from 370 to 450 °C, the weight loss of microcapsules is owing to the pyrolysis of PS. As a consequence, the operating temperature of microcapsules is better below 90 °C. The shell material microencapsulates the core material successfully.

**Figure 13:**
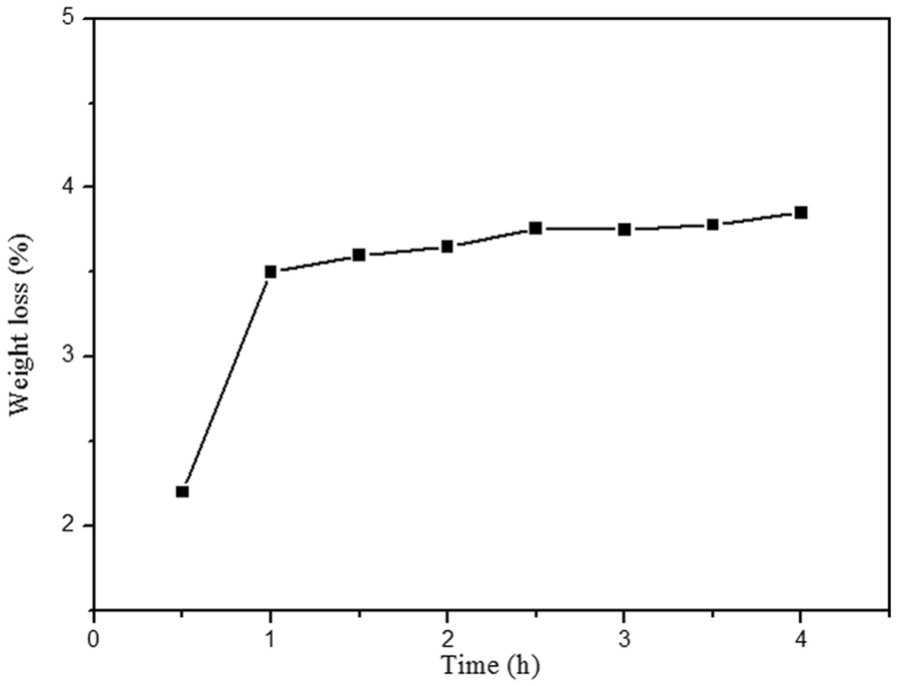
The isothermal aging experiment of DMP-30-containing microcapsules.

In order to determine the service temperature, the isothermal aging experiment of DMP-30-containing microcapsules was implemented. The curve of weight loss of microcapsules at different time kept for 50 °C is shown in Fig. 13. The weight loss of the microcapsule is about 3.55 wt% when it is placed at 50 °C for one hour. With the extension of the exposure time, the weight loss of the microcapsules decreases significantly, and the slope of the curve becomes smaller, indicating that the microcapsules can maintain the stability within 4 h of exposure at 50 °C. The weight loss before 1.5 h is due to the removal of residual water and small molecules. The weight loss of microcapsules after 1.5 h is mainly due to the slow diffusion of the core material. The weight loss of heat-treated microcapsules rises with the increase of time, which indicates that the microcapsules can not be exposed to the surrounding thermal environment for a long time, resulting in the larger weight loss of microcapsules.

**Figure 14:**
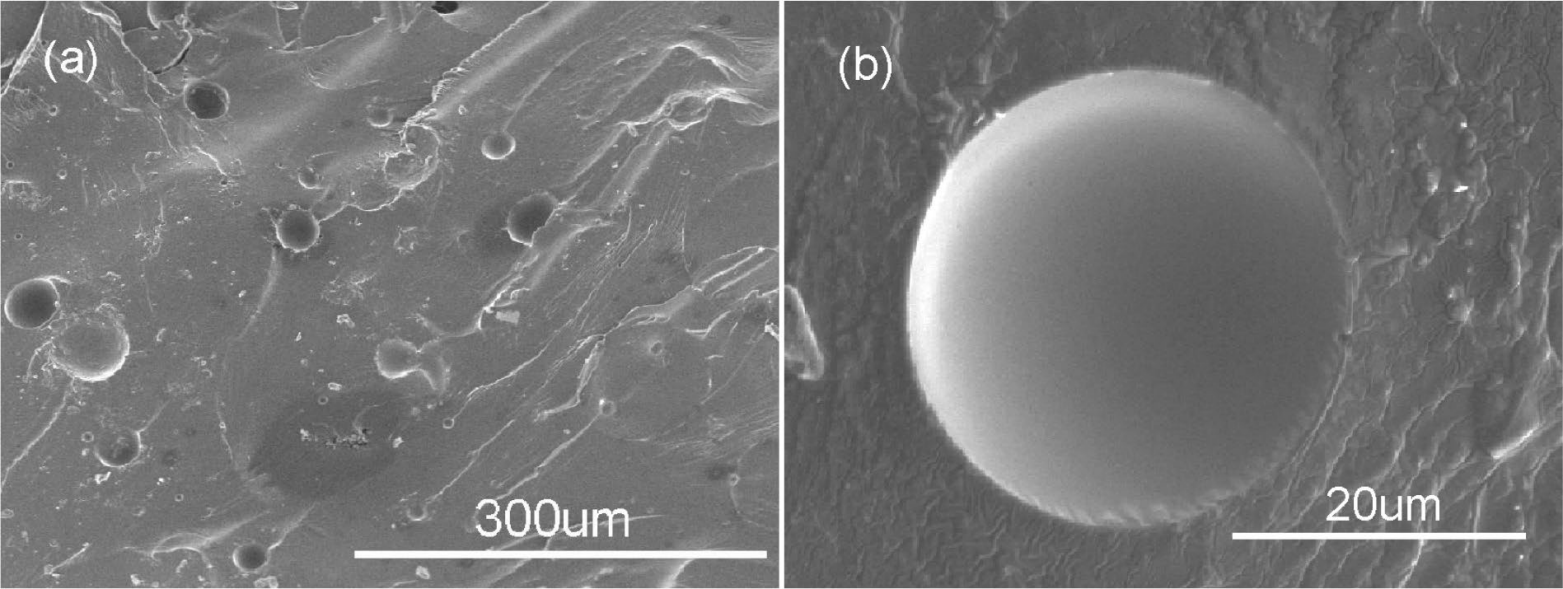
SEM photos of the fractured surface of the epoxy matrix with DMP-30-containing microcapsules.

In order to estimate the interfacial performance between the microcapsules and epoxy resin, and the integrity of microcapsules during the fabrication of composite, the SEM observation of the fractured surface of the epoxy matrix with DMP-30-containing microcapsules was carried out. From Fig. 14a, the interfacial connection between microcapsule and epoxy matrix is compact. The healing agent can be observed from fractured microcapsule in Fig. 14b, which indicates that the DMP-30-containing microcapsules can maintain integrity during the preparation of composite materials.

## Conclusion

The DMP-30-containing microcapsules with PS shell were successfully prepared via solvent evaporation technique in a W/O/W emulsion. This is achieved by forming a stable W–O–W emulsion of PS and DMP-30, evaporating oil solvent to encapsulate the core material, forming microcapsules with the dispersion function of the third phase. The influence of preparation conditions of microcapsules was investigated systematically. Finally, the optimum preparation parameters are concluded: 1.4:1 for the weight ratio of core/shell material, 250–300 rpm for the agitation rate, and 4wt% for emulsifier composed of SDS and Arabic gum (weight ratio of SDS and Arabic gum is 3:1). The chemical structure of microcapsules and reaction activity of core material in microcapsules are confirmed. The resultant microcapsules possess a smooth surface and fewer pores. The average shell thickness of microcapsules is 3.2 μm. The size distribution of microcapsules is in a range of 2–40 µm and the average diameter is 17 µm. The operating temperature of microcapsules is better below 90 °C from TGA results. The resultant microcapsules expose to 50 °C can maintain well in 4 h. The resultant microcapsules are adequate for fabricating self-healing composites. This work may be a benefit for preparing novel amine-containing microcapsules.

## Data Availability

The data used to support the findings of this study are available from the corresponding author upon request.
